# Status of knowledge, attitude and practice of poststroke dysphagia in neurological nurses in China: A cross-sectional study

**DOI:** 10.1371/journal.pone.0284657

**Published:** 2023-04-21

**Authors:** Rui Wang, Yuqing Song, Yueyue He, Shiyan Long, Ling Feng

**Affiliations:** 1 Department of Neurology, West China Hospital, Sichuan University/ West China School of Nursing, Sichuan University, Chengdu, PR China; 2 West China School of Nursing/ West China Hospital, Sichuan University, Chengdu, PR China; Universita degli Studi di Roma La Sapienza, ITALY

## Abstract

**Objectives:**

To explore the status and related factors of knowledge, attitude, and practice (KAP) of poststroke dysphagia among neurological nurses in China.

**Methods:**

Neurological nurses from 40 tertiary hospitals in Southwest China were invited to complete a survey on the knowledge, attitude, and practice of the nursing of poststroke dysphagia. We used a questionnaire to collect the participants’ information including the basic characteristics and the KAP Questionnaire on poststroke dysphagia in the neurological ward. A sample of 707 participants completed the survey.

**Results:**

The knowledge, attitude, and practice scores for the nursing of poststroke dysphagia were 12.00±4.09, 71.99±11.00, 52.22±9.08, respectively. The total score of knowledge towards the nursing of poststroke dysphagia was significantly different among nurses with different ages, working time of nursing, working time of nursing in neurology, the highest level of education, professional title, position, the method of training, the number of dysphagia-related nursing trainings, the total length of dysphagia nursing training, and the nursing basis of patients with dysphagia. The total score of attitudes towards the nursing of poststroke dysphagia was significantly different among nurses with the way they were trained, and the nursing basis for patients with dysphagia. The total score of practice towards poststroke dysphagia was significantly different among nurses with the number of dysphagia-related nursing trainings, the total length of dysphagia nursing training, the training method, and the basis of nursing patients with dysphagia.

**Conclusion:**

Neurological nurses’ knowledge score in poststroke dysphagia is relatively low, and the knowledge level needs improvement. The attitude and practice score of neurological nurses in dysphagia after stroke was much better than the knowledge score. Managers and nursing researchers should develop and offer effective training for neurological nurses to improve their knowledge, attitude and practice towards poststroke dysphagia, and then improve patients’ health outcomes.

## Introduction

Stroke is the leading cause of death in China [1]. Stroke is often accompanied by a variety of complications that affect the prognosis and quality of life of patients [2]. Dysphagia is a common consequence of stroke and a risk factor for aspiration pneumonia, which is associated with higher rates of death and disability [3]. Approximately 50%-67% of stroke patients have poststroke dysphagia [4, 5]. Early evaluation of swallowing function in stroke patients informs decisions regarding nutritional management and may reduce stroke associated pneumonia (SAP) complications [6, 7]. Patients with dysphagia (poststroke) are more at risk of developing pneumonia (22.9%), which is most likely aspiration-related than those without dysphagia (1.1%) [8]. The incidence of pneumonia may increase by 1% per day when identification is delayed [9].

Neurological nurses, as the first health care providers of poststroke patients, are more readily available than doctors and rehabilitation therapists, and play a crucial role in the multidisciplinary cooperative management of poststroke dysphagia [10]. The status of nurses’ knowledge, nursing attitude, and nursing behaviour regarding dysphagia directly affects their recognition and management of poststroke dysphagia. This not only has the potential to reduce the time for screening but can also reduce pneumonia rates and hospital length of stay, especially when coupled with appropriate early intervention [7].

Dysphagia management should be valid, reliable and repeatable. Thus, some recommendations for the clinical management of stroke patients with dysphagia have been given in the United Kingdom, the United States and Canada Stroke Guidelines [2, 11, 12]. Wang et al. [13] revealed that patients suspected of having dysphagia problems should be screened, and nurses usually perform meticulous management after screening. In recent years, some areas of China have trained nurses’ specializing in dysphagia [14, 15]. However, it is not clear whether nurses have the qualifications to screen for dysphagia. Recently, some studies on the screening and evaluation of dysphagia in stroke patients have mainly focused on evaluating and improving the screening tools and summarizing the evidence of screening methods, but no published articles explored the current status of screening and evaluation of dysphagia. Pierpoint et al. [16] found that nurses have insufficient ability to identify and manage the symptoms, signs and complications of dysphagia. Thus, it is necessary to investigate the status of nursing knowledge, attitude and practice of dysphagia after stroke, which can provide a basis for managers and educators to carry out relevant training, improve the quality of nursing and ensure the safety of patients.

The concept of knowledge, attitude and practice is a new medical theory, that takes behaviour change as the purpose, knowledge as the premise, belief and attitude as the basic driving force, and finally realizes behaviour change. It has been widely applied in many fields of medical care. In this study, we conducted a cross-sectional survey and aimed to investigate the status of nurses’ knowledge, attitude and practice in nursing for dysphagia after stroke and analyse the factors that influence the scores.

## Methods

### Study design

Our study was a cross-sectional study design, and was conducted from February to May 2021. This study was in accordance with the Strengthening the Reporting of Observational Studies (STROBE) in Epidemiology recommendations.

### Patient recruitment

We used the convenience sampling method to recruit nurses from 40 hospitals in Southwest China, including Sichuan, Chongqing, Yunnan, and Guizhou. The inclusion criteria were as follows: (1) having obtained a nurse qualification certificate; (2) working in the Department of Neurology and managing stroke patients with dysphagia; (3) clinical front-line nursing staff; and (4) voluntarily participating in this study. The exclusion criteria were as follows: (1) nursing students or nurses entrusted by other hospitals for training; (2) personnel who were mainly engaged in teaching and scientific research; and (3) not completing all questionnaire information.

### Sample size calculation

On the basis of Kline ’s guidelines, the minimum value of frequencies should be greater than 10 times the number of predicters [17, 18]. The number of independent variables in this questionnaire was 55, so the minimum sample size was 550. With the addition of a 20% non-response rate, the final sample size required was 660. We included 707 participants in this study.

### Instrument

We used online questionnaires to collect participant information. including characteristics of samples and the questionnaire “Knowledge, attitude and practice of neurological nurses on poststroke dysphagia” designed by the researchers. The researchers tested the reliability and validity of the knowledge, attitude and practice of the questionnaire. Fifty neurology nurses were invited to participate in a preliminary survey, and tested the reliability and validity of the questionnaire. The Kronbach coefficient of the knowledge questionnaire was 0.686, and the value obtained by KMO test was 0.744. The Kronbach’s α coefficient of the attitude questionnaire was 0.980, and the value of KMO test was 0.968. The Kronbach coefficient of the behavioral questionnaire was 0.945, and the value obtained by KMO test was 0.929. The Kronbach coefficient of the whole questionnaire is 0.821, and the value obtained by KMO test is 0.857. The overall reliability and validity of the scale was good.

### Basic characteristics of participants

The researcher formulated the basic characteristics of the participants according to the purpose of the investigation.

#### Questionnaire on knowledge, practice and practice

The Questionnaire on Knowledge, Attitude and Practice was developed by our research team. The questionnaire was formed based on the theory of “knowledge, attitude and practice (KAP)”. First, literature retrieval was conducted by 2 nurses with master’s degrees in stroke research, and the relevant guidelines for the management of dysphagia, expert consensus, knowledge, behaviour and attitude scales of dysphagia were mainly searched to complete the construction of the questionnaire item pool. Then 5 experts were invited (2 chief physicians of the neurology department of third-grade A hospital, 2 chief technicians of the rehabilitation department, and 1 chief nurse of nursing expert) to select and modify the items through 2 rounds of expert consultation, and finally form a questionnaire. Finally, the questionnaire consisted of 55 items with 3 parts.

#### Knowledge of neurological nurses about dysphagia after stroke

This part investigates the knowledge level of neurological nursing in stroke dysphagia, with a total of 27 items, including four templates: dysphagia screening, clinical symptoms of dysphagia, nutritional assessment and feeding management, complications, and treatment. Options were set for each item separately and 1 point and 0 points were assigned according to the right and wrong options. The total score ranges from 0 to 27. A higher score indicates a better level of knowledge regarding poststroke dysphagia. In this study, the Cronbach’s alpha coefficient for the knowledge scale was 0.686.

#### Attitudes of neurological nurses regarding poststroke dysphagia

This part evaluates neurological nurses’ attitudes towards poststroke dysphagia, and consists of 16 items. Each item is scored on a 5-point Likert scale (1 = strongly disagree; 2 = Disagree; 3 = General agreement; 4 = agree; 5 = very agree), and the total score of this part ranges from 0 to 80. A higher score reflects a better nursing belief about poststroke dysphagia in nurses. In this study, the Cronbach’s alpha coefficient for the attitudes scale was 0.980.

#### Practice of neurological nurses in poststroke dysphagia

This part evaluates neurological nurses’ practice level towards poststroke dysphagia, and includes 12 items. Each item is scored on a 5-point Likert scale (1 = never; 2 = occasionally; 3 = all the time; 4 = often; 5 = always), with a total score ranging from 0 to 60. A higher score indicates better nursing practice regarding poststroke dysphagia. In this study, the Cronbach’s alpha coefficient for the practice scale was 0.945.

### Data collection

Data collection in our study was anonymous, and the specific collected items were based on the APP called “Wenjuan Xing” (www.wjx.cn). We sent an invitation to these participants with an anonymous electronic survey link by WeChat, noting the research purposes. Nurses who volunteered and complied with the inclusion criteria in this survey were further invited to complete the questionnaire. Each IP address can submit the questionnaire only once to ensure the reliability of the data obtained.

### Data analysis

All data were exported from the questionnaire to SPSS 21.0 statistical software for logical error checking, and statistical analysis was conducted after all questionnaires were confirmed to be valid. Data with a normal distribution and homogeneity of variance were expressed as the mean ± standard deviation (*x* ± s). Comparisons between two groups were performed by t test, and comparisons between multiple groups were performed by analysis of variance. Enumeration data were expressed as frequencies, composition ratios (%) or percentages (%), and the χ^2^ test was used for comparisons between groups. Spearman correlation analysis was used to analyse the correlation between continuous variables (age, years of nursing work, years of working in the neurology department) and nursing knowledge, belief and practice scores of dysphagia. Univariate analysis was used for other classified independent variables. *P* < 0.05 was considered as statistically significant.

### Patient and public involvement

The patient and the public were not involved in this research design and conduct process.

## Results

We collected 715 questionnaires and excluded 8 incomplete questionnaires. Finally, 707 questionnaires were included for data analysis. Of these participants, 695 (98.30%) were female, and the mean age was 31.02±6.00 years. All nurses were from tertiary hospitals, the average working time was 9.47±6.49 years, and the average working time in neurology was 6.95±5.46 years. For geographic distribution, all of the nurses were from the southwest region of China. Other characteristics are shown in Table 1.

**Table 1:** General characteristics of participants (n = 707).

Variables		Item score	(%)
**Gender**	Male	12	1.70
	Female	695	98.30
**Entry-level of nursing education**	Below junior college	153	21.64
	Junior college	354	50.07
	Undergraduate	196	27.72
	Master and above	4	0.57
**Highest level of education**	Below junior college	2	0.28
	Junior college	144	20.37
	Undergraduate	553	78.22
	Master and above	8	1.13
**Professional title**	Junior nurse	110	15.56
	nurse	393	55.59
	Senior nurse	169	23.90
	Associate professor	33	4.67
	professor	2	0.28
**Position (multiple choice)**	management	46	6.51
	education	22	3.11
	clinical	643	90.95
**Number of trainings on dysphagia (including online training) (time)**	None	72	10.18
	1–3	389	55.02
	4–5	140	19.80
	5–10	53	7.50
	>10	53	7.50
**Time of training on dysphagia (including online training) (h)**	None	73	10.33
	1–3	350	49.50
	4–5	143	20.23
	5–10	63	8.91
	>10	78	11.03
**Whether to obtain dysphagia specialty nurse certificate**	Yes	13	1.84
	No	694	98.16
**If “yes”, the way you get trained is (multiple choice)**	Organized by the department	580	82.15
	Organized by the hospital	191	27.02
	Organized by social (including commission, company, etc)	141	19.94
	Self-study on the internet and literature	231	32.67
	Continuing education, further study, etc	182	25.74
**Nursing basis for patients with dysphagia is (multiple choice)**	One’s/own experience	443	62.66
	Hospital/department regulations	481	68.03
	Network/literature introduction of measures or programs	465	65.77
	Guidelines/Consensus	449	63.51
	Does not matter	6	0.85

### The status of KAP of neurological nurses in poststroke dysphagia

The score of knowledge of poststroke dysphagia was 12.00±4.09, the attitude score of poststroke dysphagia was 71.99±11.00, and the practice score of poststroke dysphagia was 52.22±9.08.

### The status of knowledge of neurological nurses in dysphagia

Regarding the knowledge of neurological nurses in poststroke dysphagia, a total of 27 items were included in four templates, including screening for dysphagia, clinical manifestations of dysphagia, nutritional assessment and food management, and management of complications. The average item score was 0.44±0.50. The scores of screenings for dysphagia were 0.22±0.42, and the scores of clinical manifestations were 0.80±0.40. The scores for nutrition assessment and food management were 0.23±0.42, and the scores for complication management were 0.64±0.48. The scores for each item are listed in the appendix (S1 Table).

### The status of attitude of neurological nurses in dysphagia

Regarding the attitude of neurological nurses in poststroke dysphagia, the item average score was 4.50±0.79. The specific scores for each item are listed in the appendix (S2 Table).

### The status of practice of neurological nurses in dysphagia

Regarding the practice of neurological nurses in dysphagia, the item average score was 4.35±0.98. The response to the statement showed that the three items with lower scores were the Items: Each newly admitted stroke patient was screened for swallowing function, Patients with dysphagia were screened daily for swallowing function, The swallowing function results of stroke patients were recorded daily, these three items all involved evaluation frequency and evaluation scope. The scores for each item are listed in the appendix (S3 Table).

### Factors associated with the status quo of KAP toward neurological nurses (monofactor analysis)

The KAP score was set as the dependent variable and the basic information was set as the independent variable. Independent variables included categorical and continuous variables. Spearman correlation analysis was performed between continuous independent variables and dependent variables (Table 2). A one-way analysis of variance was performed between the dependent variables (Table 3).

**Table 2:** Factors associated with the status of KAP (continuous variables).

Variables	The status quo of knowledge	The status quo of attitude	The status quo of practice
**Age**			
Correlation coefficient	0.100**	-0.050	-0.048
*P*	0.008	0.181	0.205
**Working time of nursing**			
Correlation coefficient	0.091*	-0.035	-0.019
*P*	0.015	0.359	0.609
**Working time of nursing in neurology**			
Correlation coefficient	0.143**	-0.019	-0.004
*P*	0.000	0.608	0.906

Note

* 0.05 > p-value > = 0.01.

**0.01 > p-value > = 0.001.

**Table 3:** Factors associated with the status quo of KAP (categorical variables).

Variables		The status quo of knowledge	The status quo of attitude	The status quo of practice
**Gender**	Male	10.83±4.345	69.67±13.963	51.33±10.722
	Female	12.02±4.090	72.03±10.547	52.24±9.062
	F	0.986	0.547	0.117
	*P*	0.321	0.460	0.732
**Highest level of education**	Secondary education	14.50±2.121	80.00±0.001	51.50±0.707
	Junior college	11.24±3.796	71.67±9.179	53.29±7.822
	bachelor degree	12.13±4.145	71.99±11.487	51.92±9.399
	Master’s degree	16.00±2.777	96.13±9.377	54.50±8.350
	F	4.683	0.722	1.048
	*P*	0.003	0.510	0.371
**Professional title**	Junior nurse	10.95±3.831	71.61±9.377	53.34±7.676
	nurse	11.79±4.054	72.20±10.962	52.20±8.867
	Senior nurse	12.68±4.115	71.57±12.461	51.12±10.662
	Associate professor	14.42±3.977	72.58±8.700	54.03±6.507
	professor	13.50±4.950	79.00±1.414	60.00±<0.001
	F	6.410	0.354	1.739
	*P*	<0.001	0.841	0.140
**Position (management)**	Yes	14.22±3.794	73.20±9.765	51.54±9.237
	No	11.84±4.072	71.91±11.079	52.27±9.079
	F	14.751	0.587	0.275
	*P*	<0.001	0.444	0.600
**Position (education)**	Yes	14.45±4.160	71.68±13.830	50.14±11.269
	No	11.92±4.072	72.00±10.906	52.29±9.008
	F	8.264	0.018	1.199
	*P*	0.004	0.892	0.274
**Number of trainings on dysphagia (including online training) (time)**	None	9.78±3.746	71.61±10.625	49.68±10.210
	1–3	11.83±4.129	71.09±11.811	51.49±9.751
	4–5	12.59±3.920	73.31±8.646	53.39±7.686
	5–10	13.74±3.933	72.49±11.157	53.09±6.940
	>10	12.94±3.559	75.19±10.037	57.09±4.588
	F	9.755	2.341	6.768
	P	<0.001	0.054	<0.001
**Time of training on dysphagia (including online training) (h)**	None	9.90±3.874	71.70±10.577	49.77±10.166
	1–3	11.53±4.103	71.32±10.941	51.75±9.546
	4–5	12.76±3.742	71.49±12.501	52.97±7.408
	5–10	13.81±4.169	74.21±6.844	53.59±8.907
	>10	13.21±3.627	74.42±11.088	54.17±8.294
	F	12.717	2.015	6.768
	P	<0.001	0.091	0.000
**The way you get trained (organized by the hospital)**	Yes	12.09±4.104	73.99±8.547	54.43±6.867
	No	11.96±4.094	71.25±11.695	51.41±9.657
	F	0.147	8.756	15.828
	P	0.702	0.003	<0.001
**The way you get trained (organized by social)**	Yes	13.33±3.813	74.06±8.630	53.57±7.166
	No	11.67±4.098	71.48±11.459	51.89±9.478
	F	19.035	6.241	3.870
	P	<0.001	0.013	0.050
**The way you get trained (Self-study on the internet and literature)**	Yes	12.97±3.864	73.15±11.281	53.28±8.229
	No	11.53±4.123	71.43±10.823	51.71±9.437
	F	19.877	3.786	4.677
	P	<0.001	0.052	0.031
**The way you get trained (Continuing education, further study, etc)**	Yes	12.78±4.130	73.35±10.632	53.12±8.493
	No	11.73±4.050	71.53±11.091	51.91±9.268
	F	9.068	3.718	2.396
	P	0.003	0.054	0.122
**Nursing basis (One’s/own experience)**	Yes	12.27±4.075	72.13±10.584	51.99±9.169
	No	11.53±4.092	71.76±11.672	52.62±8.944
	F	5.424	0.189	0.792
	P	0.020	0.664	0.374
**Nursing basis (Hospital/department regulations)**	Yes	12.51±4.053	72.56±10.011	52.97±8.102
	No	10.92±3.978	70.80±12.788	50.64±10.734
	F	23.921	3.929	10.204
	P	<0.001	0.048	0.001
**Nursing basis (Network/literature introduction of measures or programs)**	Yes	12.66±4.004	73.36±10.035	53.50±7.981
	No	10.71±3.965	69.37±12.239	49.78±10.488
	F	37.984	21.606	27.697
	P	<0.001	<0.001	<0.001
**Nursing basis (Guidelines/Consensus)**	Yes	12.71±3.954	73.10±9.795	53.23±8.565
	No	10.75±4.041	70.07±12.612	50.47±9.693
	F	39.899	12.650	15.463
	P	<0.001	<0.001	<0.001

Note: This table only presents the positive part of the factor analysis. Check the completed data in the S4 Table.

The results showed that the total score of knowledge for neurological nurses regarding dysphagia was significantly different among nurses with different ages, working time of nursing, working time of nursing in neurology, highest level of education, professional title, position, the way they received training, number of trainings related to dysphagia, total length of training for dysphagia, and nursing basis for patients with dysphagia. The total score of attitudes in neurological nurses towards dysphagia was significantly different among nurses with regard to the way they received training and the nursing basis for patients with dysphagia. The total score of practice for neurological nurses towards dysphagia was significantly different among nurses with regard to the number of dysphagia-related trainings, the total length of dysphagia training, the way they were trained, and the nursing basis of patients with dysphagia.

### Factors affecting KAP of neurological nurses in dysphagia (Multivariate analysis)

The KAP scores of neurological nurses in dysphagia were taken as the dependent variable, and factors showing significance in general data were taken as independent variables. Multiple stepwise regression analysis was conducted according to the levels of α = 0.05 in the entry model and α = 0.10 in the exit model. After the final entry into the equation, we analyze the factors affecting knowledge, attitude and practice respectively (Tables 4–6).

**Table 4:** Regression analysis of knowledge of neurological nurses in dysphagia.

Independent variables	*B*	*SE*	*β*	*t*	*P*
Constant	47.231	.560		84.291	.000
Time of training on dysphagia	-.395	.139	-.109	-2.839	.005
Nursing basis (Guidelines/Consensus)	-.982	.331	-.116	-2.962	.003
Nursing basis (Hospital/department regulations)	-1.381	.315	-.157	-4.383	.000
Professional title	-.879	.197	-.163	-4.465	.000
Nursing basis (Network/literature introduction of measures or programs)	-1.087	.335	-.126	-3.247	.001

Note: SE = Standard Error.

**Table 5:** Regression analysis of attitude of neurological nurses in dysphagia.

Independent variables	*B*	*SE*	*β*	*t*	*P*
Constant	69.062	.709		97.341	.000
Nursing basis (Network/literature introduction of measures or programs)	3.637	.873	.157	4.166	.000
The way you get trained (organized by the hospital)	2.001	.933	.081	2.145	.032

Note: SE = Standard Error.

**Table 6:** Regression analysis of practice of neurological nurses in dysphagia.

Independent variables	*B*	*SE*	*β*	*t*	*P*
Constant	46.567	.973		47.853	.000
Nursing basis (Network/literature introduction of measures or programs)	2.663	.732	.139	3.638	.000
Number of trainings on dysphagia	1.010	.348	.114	2.904	.004
Nursing basis (Hospital/department regulations)	1.452	.723	.075	2.007	.045
The way you get trained (organized by the hospital)	1.562	.790	.076	1.976	.049

Note: SE = Standard Error.

## Discussion

Our study evaluated neurological nurses’ knowledge, attitudes and behavioural levels and their associated factors. The score of knowledge of dysphagia after stroke in neurological nurses was 12.00±4.09, much lower than the results found by Knight et al. [19]. However, the dysphagia attitude score was 71.99±11.00 and the score of clinical behaviour of dysphagia was 52.22±9.08. Our score is higher than the MA Keke [20] and Sun Qian [21] results. Dysphagia rates after stroke can be as high as 50% to 67%, To manage dysphagia after stroke, it is important for nurses to have good knowledge for early identification and avoidance of unfavourable outcomes such as malnutrition, aspiration, pneumonia and death. A study in South Africa found that nurses identified limited staff and time and insufficient knowledge and training on dysphagia as barriers to appropriate dysphagia management [22]. The same results can be seen in the current study, and our results showed that only 13 (1.84%) stroke nurses had obtained the diploma of the specialized nurse in dysphagia. In addition, there was a serious shortage of training on knowledge related to dysphagia. Among 707 stroke nurses, 10.18% had not received training related to dysphagia, 55.02% had received training 1–3 times, and only 7.5% had received training > 10 times. In terms of training time, 59.83% of nurses’ training time was less than 3 h, and only 11.03% of nurses’ training time was > 10 h. From the perspective of training methods, most of the training was organized by departments, and the out-of-hospital training (including societies, companies, etc.) was only 19.94%, indicating that departments paid more attention to the management of dysphagia after stroke at the hospital level, but not enough attention from the social level. To enable stroke patients with dysphagia to receive more professional evaluation and effective guidance, it is necessary to strengthen the training of nurses on knowledge related to dysphagia.

In this study, the items of knowledge of dysphagia after stroke in neurological nurses were divided into 4 templates, including screening for dysphagia, clinical manifestations of dysphagia, nutritional assessment and food management, and management of complications. The highest score was obtained in the clinical presentation part (0.80±0.402), and the scores of screening for dysphagia, nutrition assessment and food management were extremely low (0.22±0.416, 0.23±0.421). A previous study found a score of 66.7%, compared to 80% in this study. To some extent, this reflects the poor development of clinical dysphagia, and the poor grasp of nurses’ knowledge on the screening of dysphagia. The lack of an international unified dysphagia assessment scale may lead to this result [23]. Different dysphagia assessment tools may lead to different clinical outcomes. Jannini et al. [24] found that among patients who passed the dysphagia screening, none of those classified by the GLOBE-3S (the Sapienza Global Bedside Evaluation of Swallowing after Stroke) method had pneumonia, while in those screened with the traditional method, it occurred in about a third of the patients. This suggests that the next step of our work should be to develop a standard management plan for stroke patients with dysphagia with strong clinical implementation according to the national conditions, and strengthen the clinical management plan for patients with dysphagia by means of index assessment, to promote their effective clinical management.

The nursing attitude of neurologic nurses with dysphagia after stroke was more positive. It is worth noting that the nurses expressed a positive attitude towards the training of swallowing dysfunction knowledge: 63.5% of the nurses were very willing to train, 30.7% of the nurses were willing to train, 3.8% of the nurses were less willing to train, and only 2% of the nurses were unwilling/very unwilling to receive training. The item "I think the occurrence of pulmonary infection in stroke patients with dysphagia has a certain relationship with the inadequacy of nursing work" scored the lowest. Dysphagia is the main risk factor for poststroke pneumonia, and the incidence of aspiration in patients with dysphagia exceeds 40% [3]. Bray et al. [9] and Feng et al. [25] have shown that early identification of dysphagia poststroke informs decisions regarding nutritional management and may reduce pulmonary complications. The incidence of pneumonia may increase by 1% per day when the identification of dysphagia is delayed. In the future, training on the importance and necessity of dysphagia after stroke should be strengthened to promote effective clinical implementation. Second, the item "It is necessary to screen every newly admitted stroke patient for dysphagia” ranked second to last. Wang et al. [13] suggested that an effective tool should be used to evaluate the swallowing function of each stroke patient within 24 hours of admission and before each meal and drink. For these reasons, it is vital that nurses be aware of complications associated with mismanagement and have knowledge of the importance of appropriate management. The acceptance of swallowing screening by nurses should be strengthened and it should be integrated into their daily work.

This study’s results showed that neurologic nurses’ nursing behaviour towards dysphagia after stroke was moderate and needed improvement. "Shaking the head of the bed higher than or equal to 30° every time for patients with dysphagia through nasal feeding" scored higher (the first place), followed by "instructing patients to stay in sitting or semidecubi position for 30–60 minutes after the end of nasal feeding" (the second place).Meanwhile, "screening swallowing function for each newly admitted stroke patient", "screening swallowing function for patients with dysphagia every day" and "recording swallowing function results of stroke patients daily" ranked low (the last three).China has issued several guidelines and expert consensus statements on enteral nutrition [13], which can effectively achieve good clinical operability. The results of this survey also showed that the screening of swallowing function by nurses for each newly admitted stroke patient was not ideal. The screening rate of dysphagia in the acute stage of stroke in China and the rehabilitation intervention rate were 36.4% and 49.3%, respectively, which was significantly different from the 80.0%-90.0% rates in foreign practice [26]. The reasons may be related to insufficient nursing staff allocation or insufficient emphasis on screening. In addition, the swallowing function of patients in the acute stage of stroke will recover within 1 week with the remission of the disease, and Arreola V. et al. [27] found that 26% of poststroke patients showed new signs or symptoms of impaired swallowing efficacy at the follow-up visit. Thus, it is necessary to evaluate the swallowing function of patients on a daily basis and take targeted nursing measures for patients.

In this study, the score of knowledge in neurological nurses towards dysphagia varied greatly depending on different ages, working time of nursing, working time of nursing in neurology, the highest level of education, professional title, and position. A high level of education may positively influence nurses’ work attitudes and engagement [28]. Nurses’ experience in caring for patients with dysphagia affects their ability to identify and manage dysphagia, which is consistent with the findings of Rhoda [19]. Thus, understanding these variables is important in designing strategies for clinical nurses to prevent and manage dysphagia after stroke, and nursing managers should pay more attention to nurses who may have lower KAP scores.

In this study, the scores of overall KAP and three dimensions among nurses who received training related to dysphagia, especially organized by hospitals and social organizations, were at a higher level compared with others (*P* < 0.05). This shows that society and the public’s attention to dysphagia can better affect nurses’ attitudes and behaviours towards dysphagia. This finding indicated that we should improve public education and social promotion of dysphagia knowledge.

The nursing basis of clinical nurses is statistically significant for the overall KAP score and the three-dimensional scores, especially the literature, guidelines, and expert consensus, can effectively improve the scores of all dimensions of dysphagia. This suggests that clinical nursing workers should establish the concept of evidence-based nursing, abandon previous empiricism and book-based nursing ideas, and use literature, guidelines, etc., as the main source of clinical nursing work. In addition, leaders should determine a mission, vision, and strategy and aim for knowledge management and practice [29], and clinical managers and researchers should formulate feasible and clinically guiding management measures based on the national conditions and clinical practice of each country.

## Conclusion

The score of knowledge of poststroke dysphagia in neurological nurses in the neurology department was relatively low. The attitude and behaviour scores for poststroke dysphagia were relatively higher. Managers and nursing researchers should develop and offer effective training for neurological nurses to improve their knowledge level attitude and behaviour towards poststroke dysphagia and then improve patient health outcomes.

## Supporting information

S1 Table The status of knowledge of neurological nurses in dysphagia (n = 707). (DOCX)

S2 Table The status of attitude of neurological nurses in dysphagia (n = 707). (DOCX)

S3 Table The status of practice of neurological nurses in dysphagia (n = 707). (DOCX)

S4 Table Factors associated with the status quo of KAP (categorical variables). (DOCX)

S1 Data (XLSX)
