# Supplementary material for: Status of knowledge, attitude and practice of poststroke dysphagia in neurological nurses in China: A cross-sectional study
Source: PLoS One. 2023 Apr 21;18(4):e0284657. doi: 10.1371/journal.pone.0284657 (PMC10121028; doi:10.1371/journal.pone.0284657)
Supplement: S1 Table — (DOCX) [file pone.0284657.s001.docx]

supplementary table 1: The status of knowledge of neurological nurses in dysphagia（n=707）

| Variables | Average score, M± SD |
| --- | --- |
| 1. Nutritional support pathway | 0.89±0.31 |
| 2. Clinical manifestations of aspiration caused by dysphagia | 0.80±0.40 |
| 3. Methods to avoid gastrointestinal intolerance in patients with enteral nutrition | 0.72±0.45 |
| 4. The most serious complication after dysphagia | 0.64±0.48 |
| 5. Postural adjustment for patients with dysphagia | 0.62±0.49 |
| 6. Nutritional risk assessment | 0.61±0.49 |
| 7. Dehydration due to dysphagia | 0.57±0.50 |
| 8. Mouthful eating | 0.57±0.50 |
| 9. Evaluation of swallowing function screening | 0.54±0.50 |
| 10. The swallowing process associated with stroke | 0.52±0.50 |
| 11. Compensatory measures for eating in patients with dysphagia after stroke | 0.50±0.50 |
| 12. Iatrogenic risk factors for aspiration | 0.48±0.50 |
| 13. Complications of persistent indwelling of nasogastric tube | 0.48±0.50 |
| 14. Indicators for nutritional risk assessment | 0.45±0.50 |
| 15. Drinking water test in depression | 0.44±0.50 |
| 16. The manifestation of dysphagia in pharynx | 0.37±0.48 |
| 17. Risk factors for malnutrition in stroke patients | 0.33±0.47 |
| 18. Oral phase dysphagia | 0.33±0.47 |
| 19. Swallowing angiography | 0.29±0.46 |
| 20. Gold standard for swallowing function score | 0.28±0.45 |
| 21. Gugging swallowing screen | 0.27±0.44 |
| 22. Respiratory function training for patients with dysphagia | 0.25±0.43 |
| 23. Percutaneous endoscopic gastrostomy | 0.24±0.43 |
| 24. Timing of nutritional risk assessment in stroke patients | 0.23±0.42 |
| 25. Duration of swallowing function screening | 0.22±0.42 |
| 26. Determination of nasogastric tube position | 0.19±0.39 |
| 27. Swallowing function screening tool | 0.15±0.36 |

Note: the results in the list are arranged according to the score, which is inconsistent with the items in the questionnaire
