# Supplementary material for: Status of knowledge, attitude and practice of poststroke dysphagia in neurological nurses in China: A cross-sectional study
Source: PLoS One. 2023 Apr 21;18(4):e0284657. doi: 10.1371/journal.pone.0284657 (PMC10121028; doi:10.1371/journal.pone.0284657)
Supplement: S2 Table — (DOCX) [file pone.0284657.s002.docx]

supplementary table 2: The status of attitude of neurological nurses in dysphagia（n=707）

| Variables | Average score, M± SD |
| --- | --- |
| 1. I think the choice of body position is very important for stroke patients with dysphagia when eating | 4.59±0.75 |
| 2. I think it is very important to guide or help patients with dysphagia to recover their swallowing function | 4.58±0.77 |
| 3. I think it is very important for stroke patients with dysphagia to keep the head of the bed shaking height ≥30° during nasal feeding | 4.58±0.75 |
| 4. I think the choice of food type, character and texture is very important for stroke patients with dysphagia | 4.58±0.75 |
| 5. I think maintaining good oral hygiene is very important to prevent aspiration pneumonia in stroke patients with dysphagia | 4.58±0.73 |
| 6. I think it is important to teach patients and their families to recognize signs and complications of dysphagia | 4.55±0.77 |
| 7. I think it’s important to screen every stroke patient for dysphagia | 4.55±0.75 |
| 8. I would like to receive training in the nursing of post stroke dysphagia | 4.54±0.73 |
| 9. I think it is important to assist patients with dysphagia in choosing appropriate food compensation measures | 4.51±0.77 |
| 10. I think there is a need for a multidisciplinary team to come up with disciplinary recommendations to help patients with dysphagia | 4.51±0.75 |
| 11. I think early screening for dysphagia is important | 4.50±0.79 |
| 12. I think the ability of nurses to recognize and manage dysphagia in stroke patients is important | 4.50±0.76 |
| 13. I think other medical professionals can help patients with dysphagia | 4.42±0.80 |
| 14. I think stroke dysphagia patient tableware choice is very important | 4.41±0.82 |
| 15. I think it is important to record the swallowing function screening results of stroke patients on a daily basis | 4.41±0.82 |
| 16. I think the occurrence of pulmonary infection in stroke patients with dysphagia and nursing work have a certain relationship | 4.17±0.98 |

Note: the results in the list are arranged according to the score, which is inconsistent with the items in the questionnaire
