# Supplementary material for: Status of knowledge, attitude and practice of poststroke dysphagia in neurological nurses in China: A cross-sectional study
Source: PLoS One. 2023 Apr 21;18(4):e0284657. doi: 10.1371/journal.pone.0284657 (PMC10121028; doi:10.1371/journal.pone.0284657)
Supplement: S3 Table — (DOCX) [file pone.0284657.s003.docx]

supplementary table 3: The status of practice of neurological nurses in dysphagia（n=707）

| Variables | Average score, M± SD |
| --- | --- |
| 1. For patients with dysphagia, the head of the bed should be ≥30° high during nasal feeding | 4.66±0.72 |
| 2. After the end of nasal feeding, the patients were instructed to remain in sitting or semidecubitus position for 30-60 minutes | 4.62±0.76 |
| 3. Guide and help stroke patients with dysphagia to maintain good oral hygiene habits | 4.62±0.74 |
| 4. Confirmation of duct position before nasal feeding | 4.54±0.88 |
| 5. Re-evaluate the patient's swallowing function as the condition changes | 4.35±0.93 |
| 6. Inform each stroke patient with dysphagia about the method of eating posture compensation | 4.34±0.93 |
| 7. Explain the knowledge of dysphagia to the patient/family | 4.34±0.89 |
| 8. Guide or help the patient to prepare food and change the food characteristics and texture according to the level of dysphagia (e.g., using thickener) | 4.30±0.96 |
| 9. To instruct patients with dysphagia to recover their deglutition function | 4.26±0.98 |
| 10. Each newly admitted stroke patient was screened for swallowing function | 4.25±1.05 |
| 11. Patients with dysphagia were screened daily for swallowing function | 4.02±1.21 |
| 12. The swallowing function results of stroke patients were recorded daily | 3.92±1.29 |

Note: the results in the list are arranged according to the score, which is inconsistent with the items in the questionnaire
