# Supplementary material for: Status of knowledge, attitude and practice of poststroke dysphagia in neurological nurses in China: A cross-sectional study
Source: PLoS One. 2023 Apr 21;18(4):e0284657. doi: 10.1371/journal.pone.0284657 (PMC10121028; doi:10.1371/journal.pone.0284657)
Supplement: S4 Table — (DOCX) [file pone.0284657.s004.docx]

supplementary table 4: Factors associated with the status quo of KAP (categorical variables)

| Variables | | The status quo of knowledge | The status quo of attitude | The status quo of practice |
| --- | --- | --- | --- | --- |
| **Gender** | Male | 10.83±4.345 | 69.67±13.963 | 51.33±10.722 |
|  | Female | 12.02±4.090 | 72.03±10.547 | 52.24±9.062 |
|  | F | 0.986 | 0.547 | 0.117 |
|  | *P* | 0.321 | 0.460 | 0.732 |
| **Entry level of nursing education** | Below junior college | 11.88±4.040 | 70.73±10.085 | 51.61±8.343 |
|  | Junior college | 11.86±3.967 | 72.39±10.927 | 52.71±8.829 |
|  | Undergraduate | 12.27±4.361 | 72.20±11.818 | 51.82±10.034 |
|  | Master and above | 15.50±2.380 | 75.25±7.544 | 51.75±10.905 |
|  | F | 1.433 | 0.973 | 0.705 |
|  | *P* | 0.232 | 0.405 | 0.549 |
| **Highest level of education** | Secondary education | 14.50±2.121 | 80.00±0.001 | 51.50±0.707 |
|  | Junior college | 11.24±3.796 | 71.67±9.179 | 53.29±7.822 |
|  | bachelor degree | 12.13±4.145 | 71.99±11.487 | 51.92±9.399 |
|  | Master's degree | 16.00±2.777 | 96.13±9.377 | 54.50±8.350 |
|  | F | 4.683 | 0.722 | 1.048 |
|  | *P* | 0.003 | 0.510 | 0.371 |
| **Professional title** | Junior nurse | 10.95±3.831 | 71.61±9.377 | 53.34±7.676 |
|  | nurse | 11.79±4.054 | 72.20±10.962 | 52.20±8.867 |
|  | Senior nurse | 12.68±4.115 | 71.57±12.461 | 51.12±10.662 |
|  | Associate professor | 14.42±3.977 | 72.58±8.700 | 54.03±6.507 |
|  | professor | 13.50±4.950 | 79.00±1.414 | 60.00±＜0.001 |
|  | F | 6.410 | 0.354 | 1.739 |
|  | *P* | ＜0.001 | 0.841 | 0.140 |
| **Position（management）** | Yes | 14.22±3.794 | 73.20±9.765 | 51.54±9.237 |
|  | No | 11.84±4.072 | 71.91±11.079 | 52.27±9.079 |
|  | F | 14.751 | 0.587 | 0.275 |
|  | *P* | ＜0.001 | 0.444 | 0.600 |
| **Position（education）** | Yes | 14.45±4.160 | 71.68±13.830 | 50.14±11.269 |
|  | No | 11.92±4.072 | 72.00±10.906 | 52.29±9.008 |
|  | F | 8.264 | 0.018 | 1.199 |
|  | *P* | 0.004 | 0.892 | 0.274 |
| **Position（clinical）** | Yes | 11.93±4.096 | 71.98±11.116 | 52.30±9.057 |
|  | No | 12.70±4.042 | 72.11±9.790 | 51.42±9.391 |
|  | F | 2.096 | 0.008 | 0.548 |
|  | P | 0.148 | 0.930 | 0.460 |
| **Number of trainings on dysphagia (including online training)（time）** | None | 9.78±3.746 | 71.61±10.625 | 49.68±10.210 |
|  | 1-3 | 11.83±4.129 | 71.09±11.811 | 51.49±9.751 |
|  | 4-5 | 12.59±3.920 | 73.31±8.646 | 53.39±7.686 |
|  | 5-10 | 13.74±3.933 | 72.49±11.157 | 53.09±6.940 |
|  | ＞10 | 12.94±3.559 | 75.19±10.037 | 57.09±4.588 |
|  | F | 9.755 | 2.341 | 6.768 |
|  | P | ＜0.001 | 0.054 | ＜0.001 |
| **Time of training on dysphagia (including online training)（h）** | None | 9.90±3.874 | 71.70±10.577 | 49.77±10.166 |
|  | 1-3 | 11.53±4.103 | 71.32±10.941 | 51.75±9.546 |
|  | 4-5 | 12.76±3.742 | 71.49±12.501 | 52.97±7.408 |
|  | 5-10 | 13.81±4.169 | 74.21±6.844 | 53.59±8.907 |
|  | ＞10 | 13.21±3.627 | 74.42±11.088 | 54.17±8.294 |
|  | F | 12.717 | 2.015 | 6.768 |
|  | P | ＜0.001 | 0.091 | 0.000 |
| **Whether to obtain deglutition disorder specialty nurse certificate** | Yes | 11.31±3.816 | 73.31±10.672 | 55.23±7.049 |
|  | No | 12.01±4.101 | 71.97±11.008 | 52.17±9.113 |
|  | F | 0.375 | 0.189 | 1.452 |
|  | P | 0.540 | 0.664 | 0.229 |
| **The way you get trained（organized by the department）** | Yes | 12.26±4.043 | 72.22±10.538 | 52.67±8.725 |
|  | No | 10.83±4.133 | 70.87±12.906 | 50.09±10.367 |
|  | F | 12.763 | 1.563 | 8.479 |
|  | P | ＜0.001 | 0.212 | 0.004 |
| **The way you get trained（organized by the hospital）** | Yes | 12.09±4.104 | 73.99±8.547 | 54.43±6.867 |
|  | No | 11.96±4.094 | 71.25±11.695 | 51.41±9.657 |
|  | F | 0.147 | 8.756 | 15.828 |
|  | P | 0.702 | 0.003 | ＜0.001 |
| **The way you get trained（organized by social）** | Yes | 13.33±3.813 | 74.06±8.630 | 53.57±7.166 |
|  | No | 11.67±4.098 | 71.48±11.459 | 51.89±9.478 |
|  | F | 19.035 | 6.241 | 3.870 |
|  | P | ＜0.001 | 0.013 | 0.050 |
| **The way you get trained（Self-study on the internet and literature）** | Yes | 12.97±3.864 | 73.15±11.281 | 53.28±8.229 |
|  | No | 11.53±4.123 | 71.43±10.823 | 51.71±9.437 |
|  | F | 19.877 | 3.786 | 4.677 |
|  | P | ＜0.001 | 0.052 | 0.031 |
| **The way you get trained(Continuing education, further study, etc.)** | Yes | 12.78±4.130 | 73.35±10.632 | 53.12±8.493 |
|  | No | 11.73±4.050 | 71.53±11.091 | 51.91±9.268 |
|  | F | 9.068 | 3.718 | 2.396 |
|  | P | 0.003 | 0.054 | 0.122 |
| **Nursing basis(One's/own experience)** | Yes | 12.27±4.075 | 72.13±10.584 | 51.99±9.169 |
|  | No | 11.53±4.092 | 71.76±11.672 | 52.62±8.944 |
|  | F | 5.424 | 0.189 | 0.792 |
|  | P | 0.020 | 0.664 | 0.374 |
| **Nursing basis(Hospital/department regulations)** | Yes | 12.51±4.053 | 72.56±10.011 | 52.97±8.102 |
|  | No | 10.92±3.978 | 70.80±12.788 | 50.64±10.734 |
|  | F | 23.921 | 3.929 | 10.204 |
|  | P | ＜0.001 | 0.048 | 0.001 |
| **Nursing basis(Network/literature introduction of measures or programs)** | Yes | 12.66±4.004 | 73.36±10.035 | 53.50±7.981 |
|  | No | 10.71±3.965 | 69.37±12.239 | 49.78±10.488 |
|  | F | 37.984 | 21.606 | 27.697 |
|  | P | ＜0.001 | ＜0.001 | ＜0.001 |
| **Nursing basis(Guidelines/Consensus)** | Yes | 12.71±3.954 | 73.10±9.795 | 53.23±8.565 |
|  | No | 10.75±4.041 | 70.07±12.612 | 50.47±9.693 |
|  | F | 39.899 | 12.650 | 15.463 |
|  | P | ＜0.001 | ＜0.001 | ＜0.001 |
